# Detecting sources of anger in automated driving: driving-related and external factor

**DOI:** 10.3389/fnrgo.2025.1548861

**Published:** 2025-05-09

**Authors:** Jordan Maillant, Christophe Jallais, Stéphanie Dabic

**Affiliations:** ^1^Valeo BRAIN Division, Annemasse, France; ^2^LESCOT, IFSTTAR, Univ Gustave Eiffel, Univ Lyon, Lyon, France

**Keywords:** anger sources, anger detection, physiological indicators, ocular behavior, subjective evaluations, automated driving

## Abstract

**Introduction:**

Anger while driving is often provoked by on-road events like sudden cut-offs but can also arise from external factors, such as rumination of negative thoughts. With the rise of autonomous vehicles, drivers are expected to engage more in non-driving activities, potentially increasing the occurrence of anger stemming from non-driving-related sources. Given the well-established link between anger and aggressive driving behaviors, it is crucial to detect and understand the various origins of anger in autonomous driving contexts to enhance road safety.

**Methods:**

This study investigates whether physiological (cardiac and respiratory activities) and ocular indicators of anger vary depending on its source (driving-related or external) in a simulated autonomous driving environment. Using a combination of autobiographical recall (AR) for external anger induction and driving-related scenarios (DS), 47 participants were exposed to anger and/or neutral conditions across four groups.

**Results:**

The results revealed that combined anger induction (incorporating both external and driving-related sources) led to higher subjective anger ratings, more heart rate variability. However, when examined separately, individual anger sources did not produce significant differences in physiological responses and ocular strategies.

**Discussion:**

These results suggest that the combination of anger-inducing events, rather than the specific source, is more likely to provoke a heightened state of anger. Consequently, future research should employ combined induction methods to effectively elicit anger in experimental settings. Moreover, anger detection systems should focus on the overall interplay of contributing factors rather than distinguishing between individual sources, as it is this cumulative dynamic that more effectively triggers significant anger responses.

## 1 Introduction

Anger is the most studied emotion in driving (Zepf et al., 2020) because it has a detrimental impact on driving performance (Jeon et al., 2014) and visual attention (Zhang et al., 2016). Angry drivers are more likely to exhibit aggressive behavior (Mesken et al., 2007; Precht et al., 2017) and may experience delayed reaction times (Steinhauser et al., 2018). Anger can also negatively impact automated driving, resulting in reduced takeover performance (Sanghavi et al., 2020). Given the prevalence of anger and its harmful effects on driving, a lot of research has been carried out in recent years to detect (Zepf et al., 2020) and prevent or mitigate it (Braun et al., 2021). Nevertheless, the question of the source of anger is rarely addressed. This study focuses on anger detection, asking whether the source of anger is important for the detection process.

Adopting the perspective of appraisal theorists (Moors et al., 2013), anger arises as a consequence of how we evaluate a situation (Wranik and Scherer, 2010). This recursive evaluation as described in Scherer's principal components model (Scherer, 2009), is based on several key factors:

- Relevance (to my objectives).
- Implication (what does it imply for me, who are the actors and what are their motives).
- Coping (what are my reaction options).
- Normative significance (in line with my values and social norms).

For instance, anger may flare up when another driver's inappropriate behavior (implication) blocks us (coping), making us late for an important appointment (relevance). Also, when for example, a driver uses the emergency lane to overtake during a traffic jam (normative significance). Behaviors like weaving and cutting are commonly reported as major triggers of driver anger (Wickens et al., 2013). In automated driving, vehicle behavior can also influence the driver's emotional state (Alsaid et al., 2023). When given the opportunity, some drivers may take control of the vehicle to prevent anger (coping) if it does not behave as they expect (relevance; Pan et al., 2024). This tendency is especially pronounced under time pressure (implications; Techer et al., 2019).

While anger during driving often stems from the driving environment, it is not exclusive to it, and may involve external factors. In particular, ruminating on episodes of anger while driving could also be a form of anger during driving that negatively affects performance (Suhr, 2016). Furthermore, the rise of autonomous vehicles, which will encourage more non-driving activities, may introduce additional sources of anger. Consequently, anger behind the wheel can arise from a variety of sources, both related and unrelated to the driving environment.

Although theories have long diverged on the very definition of emotion (for a short review, see Thanapattheerakul et al., 2018), many agree, and it is the case in the component process model (Scherer, 2009), that emotions involve subjective feelings and physiological and behavioral responses. In autonomous driving, the behavioral aspect of driving is only observable when the driver is taking control. As a result, only physiological and subjective cues remain. Detecting physiological variations in the driver could thus provide clues to his/her emotional state. Because of large inter-individual differences, monitoring physiological variations to infer the driver's emotional state is a major challenge pursued both by industrial and academic research.

With the same aim of studying anger during driving, researchers employ various induction methods such as videos, images, music, autobiographical recall and driving scenarios (Zepf et al., 2020). Even though many of these techniques have been evaluated as suitable to induce anger (Siedlecka and Denson, 2019), they nevertheless rely on different sources of anger (inherent or not to driving). Two questions arise: Do these distinct sources of anger are accompanied by similar or different subjective and physiological responses? Does being angry before being confronted with anger-provoking road events increase the intensity of the feeling of anger and the associated physiological responses? Answering this question would help determine whether the source of anger matters in detecting anger during driving, and whether it should influence the design of future studies and driver state monitoring systems.

Most research on recognizing drivers' emotions relies on cardiac signals, followed by electrodermal and respiratory data (Zepf et al., 2020). The Autonomic Nervous System (ANS), which regulates various bodily functions, plays a critical role. It is divided into two main branches: the sympathetic and parasympathetic systems, typically associated with arousing and calming effects, respectively. Emotional arousal (whether positive or negative) activates the sympathetic system, and these changes can be observed in the cardiac and respiratory signals. As described in Li and Zheng (2022), the standard deviation of all normal to normal RR intervals (SDNN) reflects, in the time domain, the balance between sympathetic and parasympathetic activity. RR intervals correspond to the time between two heart beats [R peaks on an electrocardiogram (ECG)]. The mean of the squared successive differences between adjacent RR intervals (RMSSD) is an indicator of parasympathetic activity. In the frequency domain, high frequencies (HF) are indicative of parasympathetic activity, while low frequencies (LF) mainly reflect sympathetic activity. The LF/HF ratio thus represents the relationship between these two activities. Negative emotions, such as fear and anger, are typically linked to reduced parasympathetic activity, as indicated by lower HF and RMSSD values. The relationship between anger and sympathetic activity, measured by heart rate variability (HRV), is more nuanced and remains a topic of debate (Gullett et al., 2023).

Although general trends linking ANS responses to emotions have been observed (Kreibig, 2010), significant variations exist across studies. These variations seem to be more pronounced for studies presenting inductions unrelated to driving activity (see Table 1). Using the driving scenario (DS) to induce anger, an elevated heart rate (HR) was observed in Wan et al. (2017) and Stephens and Groeger (2011) but no variation in Wang et al. (2024) and Mesken et al. (2007). While anger is induced by non-driving related methods such as autobiographical recall (AR) or films, some authors found increased HR (FakhrHosseini and Jeon, 2019; Marci et al., 2007; Rainville et al., 2006), decrease HR (Lafont et al., 2018, 2019) or no variation (Francis et al., 2015; Wu et al., 2019). On HRV indicators, when anger is induced by DS, the anger group showed an increase in SDNN though no significant changes in RMSSD, LF, HF, and LF/HF (Wang et al., 2024). With non-driving related methods, Marci et al. (2007) reported a decrease in HF while non-significant modulation was measured in Rainville et al. (2006) and an increase is observed in Francis et al. (2015). LF was increased in Francis et al. (2015) and McCraty et al. (1995). A decrease in the LF/HF ratio alongside an augmentation of RMSSD was also observed in Lafont et al. (2019).

**Table 1:** Summary of physiological and ocular modulations depending on the source of anger in the cited articles.

**Indicators**	**Anger induction method**	
	**Related to driving**	**Unrelated to driving**
**Cardiac**		
HR	↑ (Stephens and Groeger, 2011; Wan et al., 2017) ns (Mesken et al., 2007; Wang et al., 2024)	↑ (FakhrHosseini and Jeon, 2019; Marci et al., 2007; McCraty et al., 1995; Rainville et al., 2006) ↓ (Lafont et al., 2018, 2019) ns (Francis et al., 2015; Wu et al., 2019)
SDNN	↑ (Wang et al., 2024)	↑ (Francis et al., 2015) ns (FakhrHosseini and Jeon, 2019)
RMSSD	ns (Wang et al., 2024)	↑ (Lafont et al., 2019)
LF	↑ (Wan et al., 2017) ns (Wang et al., 2024)	↑ (Francis et al., 2015; McCraty et al., 1995)
HF	ns (Wang et al., 2024)	↑ (Francis et al., 2015) ↓ (Marci et al., 2007) ns (McCraty et al., 1995; Rainville et al., 2006)
LF/HF	ns (Wang et al., 2024)	↑ (McCraty et al., 1995) ↓ (Lafont et al., 2018)
**Respiratory**		
BR	↑ (Wan et al., 2017) ns (Wang et al., 2024)	↑ (Francis et al., 2015; Rainville et al., 2006)
**Ocular**		
Saccade amplitude		↓ (Lafont et al., 2018)
Fixations	↑ fixations on front view >dashboard (Li G. et al., 2020) ↑ fixations on front view (Huo et al., 2020) ns vertical gaze variance (Zhang et al., 2016) ↓ horizontal gaze variance (Zhang et al., 2016)	↑ longer fixations duration (Pan et al., 2024) ↓ horizontal gaze variance (Pan et al., 2024)

↑ indicates a significant increase; ↓ indicates a significant decrease; ns indicates a non-significant effect.

On respiration, breath rate (BR) was higher in Wan et al. (2017) and no different in Wang et al. (2024) while regarding induction by DS. BR was higher in Francis et al. (2015) and Rainville et al. (2006) following non-driving-related induction.

In addition to physiological data, eye behavior data can provide valuable insights into a driver's emotional and attentional state (Skaramagkas et al., 2021). A major advantage for future driver-monitoring systems is that this data can be accessed by infra-red camera without obstructing the driver. Anger during driving impairs the ability to perceive potential hazards (Zhang et al., 2016; Pan et al., 2024) and delays the localization of road elements (Jallais et al., 2014). However, there is no consensus on which specific eye characteristics are the most reliable for recognizing emotions (Lim et al., 2020). Despite this, multiple studies, with more evidence for anger induced by DS, suggest a common visual pattern associated with anger. For instance, aggressive drivers have reduced horizontal visual scanning, reduced saccade amplitudes and spend less time monitoring the peripheral environment (Lafont et al., 2018; Li G. et al., 2020; Zhang et al., 2016). They also tend to fixate more on the central field of view (Huo et al., 2020). Pan et al. (2024) examined anger induction in a study where participants had to regain control of an autonomous vehicle in response to a system failure. Anger induced using a video clip and AR, led to a narrowing of visual scanning, reduced horizontal gaze variance, and prolonged fixation durations. Expanding on this, rumination on negative thoughts increases the likelihood of mind-wandering episodes (Albert et al., 2022), which in turn leads to a narrowing of visual attention while driving (He et al., 2011). Collectively, these findings suggest that anger, regardless of whether it originates from driving or unrelated factors, results in a narrowing visual scanning pattern, impairing drivers' ability to detect potential hazards. Consequently, both sources of anger appear to be associated with similar ocular behaviors.

To summarize, it would appear that anger induced by DS or AR generally increases HR and BR and leads to a narrower visual scanning. HRV data most often show increased cardiac variability, attributed to sympathetic activity (LF, LF/HF, SDNN). However, less consistent findings in HRV and little evidence from ocular behavior, are highlighted by studies inducing anger by methods unrelated to the driving environment. Finally, being confronted with irritating road events while already angry is not studied, leaving the question open. A summary of the exposed literature is provided in Table 1.

Are the physiological differences reported in these studies due to the difference in anger induction methods? What are the impacts of a combination of these methods? Are the signs of eye behavior (i.e., reduced visual field) common to sources of anger? This study aims to answer this by examining the physiological and ocular markers of anger induced by driving-related scenarios (DS) and/or external sources (Autobiographical Recall, AR) in an automated driving context. We used autonomous driving to minimize the impact of motor actions on physiological responses, recognizing that anger can also arise without controlling the vehicle (Techer et al., 2019). Subjectively, we hypothesize that both DS and AR inductions will similarly elevate subjective anger, marked by increased arousal and diminished valence and perceived control, with combined inductions producing stronger effects. Physiologically, we expect both AR and DS elevate HR and BR with differences regarding variabilities: we expect DS-induced anger provoking higher cardiac variability (increased SDNN, LF, LF/HF and reduced HF, RMSSD) and respiratory variability (reduced RMSSD) associated with excitatory activity. We further expect that the combination of induction would increase these variations. Regarding ocular data, we hypothesize that, compared to a neutral state, anger whether induced by AR or DS would lead to a decline in visual attention, characterized by reduced horizontal scanning and fewer fixations on rear-view mirrors. Finally, given the lack of consensus in the literature regarding the relationship between subjective and physiological manifestations, we aim to further investigate these connections. Specifically, we hypothesize that self-reported levels of anger and arousal will be strongly positively correlated with the HRV indicators SDNN, LF, and LF/HF. The results could inform the design of in-car systems that monitor comprehensive anger dynamics, contributing to safer driving conditions in automated vehicles.

## 2 Materials and methods

### 2.1 Participants

Fifty-three French volunteers were involved. All were Valeo's workers and had a valid driving license for at least 3 years. Six participants were removed from the analysis due to simulator issues, a poor-quality cardiac signal (>15% of missing data) or poor compliance with instructions. Participants were divided into four groups (*aa, an, na, nn*), where each group experienced different combinations of emotional induction (anger or neutral) through two techniques: Autobiographical Recall (AR) and Driving Scenario (DS). The group names reflect the specific conditions, such as “na” for neutral AR and anger DS. Thirteen participants remained in the *aa* group, 12 in *na*, 12 in *an*, and 10 in the *nn* group. A detailed composition of the groups is presented in Table 2.

**Table 2:** Composition of groups: mean age (SD) and sex distribution and experimental session assigned.

**Characteristics**	**Names of groups**			
	**aa**	**na**	**an**	**nn**
	**Associated inductions**			
	**Anger AR Anger DS**	**Neutral AR Anger DS**	**Anger AR Neutral DS**	**Neutral AR Neutral DS**
*N*	13	12	12	10
Age (SD)	34.00 (10.93)	37.97 (14.95)	38.14 (13.46)	34.72 (11.18)
Driving experience	16.08 (12.72)	19.67 (15.49)	20.41 (13.19)	15.33 (11.47)
M	8	7	7	7
F	5	5	5	3

### 2.2 Experimental design

A between-subjects factorial design was employed with Group (*aa, an, na, nn*) as the only factor.

### 2.3 Apparatus

**Figure 1:**
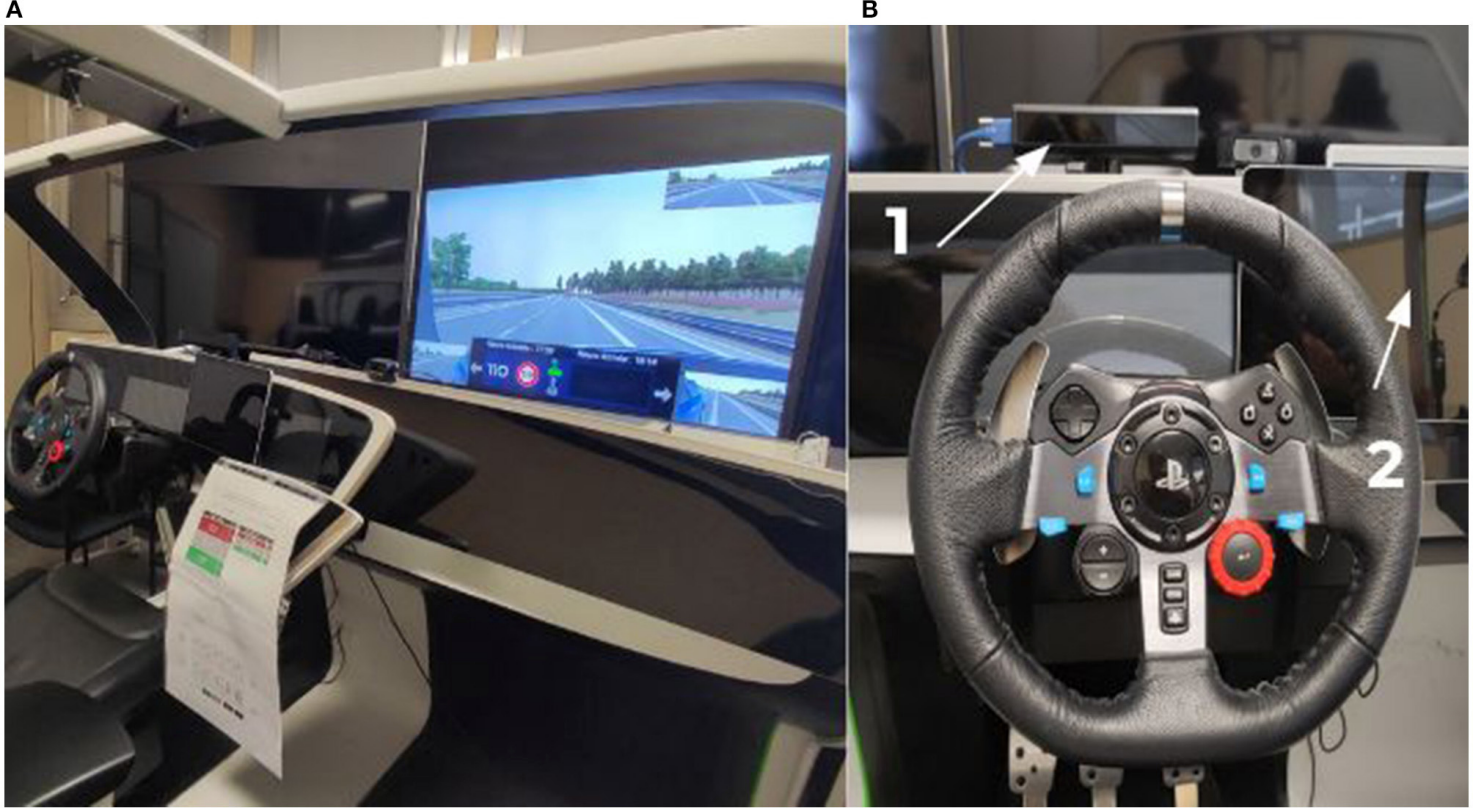
Overview of the driving simulator. Left **(A)** View of the cabin and screen, Right **(B)**, (1) Position of the eye tracker system, (2) Position of the tactile tablet used to transmit instructions and emotion questionnaires during the experiment.

Unity 3D software and a fixed-base driving simulator composed of a Logitech G29 steering wheel and pedal set were used (Figure 1). Participants were seated 220–240 cm (adjustable seat) from a 65-in. screen. BIOPAC MP160 was used to collect measurements of cardiac and respiratory signals (RSP) at a sampling rate of 500 Hz. The electrocardiogram signal (ECG) was collected from three pre-gelled electrodes (Ag-AgCl) placed on the participant's chest. The respiratory signal was collected from a respiration belt placed right under the chest. Finally, Fovio, a desktop eye tracker was used to capture ocular metrics at a sampling rate of 62 Hz.

### 2.4 Anger induction materials

#### 2.4.1 Autobiographical recall (AR)

The autobiographical recall technique consists in asking individuals to write down a personal memory in which they strongly experienced the targeted emotion. Similar to the study by Jallais and Gilet (2010), participants were given 10 min to complete this task. The participants in the anger conditions were asked to recall and write down a situation in which they had experienced anger. They were encouraged to include as many details as possible and to vividly recount the event. For the induction of a neutral emotional state, participants were asked to describe their daily routine for the same duration. Autobiographical recall was chosen because it is the method that most closely approximates the rumination of negative thoughts that individuals might experience while driving.

#### 2.4.2 Driving scenario (DS)

Participants were involved either in anger or neutral scenarios. These scenarios were designed to be comparable. Since the ego vehicle operated in autonomous mode, the speed and behavior of both the ego vehicle and surrounding vehicles in the simulation were fully controlled. Four specific events were created to provoke anger in the anger scenario. A detailed description of these events, along with their counterparts in the neutral scenario, is provided in the Supplementary material.

### 2.5 Measures

#### 2.5.1 Subjective measures

We asked participants to subjectively report their current emotional state according to both dimensional and categorical emotional scales. This double questioning allowed participants to express their emotional state in different ways, giving us a clearer picture. Also, as the autobiographical recall technique can induce other closely related states such as sadness (Mills and D'Mello, 2014), assessing individuals across multiple emotional states allows us to confirm whether the targeted emotion is indeed the predominant one felt.

##### 2.5.1.1 Dimensional scales

The Self-Assessment Manikin (SAM; Bradley and Lang, 1994) a simple visual questionnaire was employed to evaluate valence, arousal and the control dimensions of their emotional state. Definitions given to them were: “Valence, evaluate from negative (left) to positive (right) your emotional state;” “Arousal, evaluate from arouse (left) to calm (right) your emotional state;” “Control, Evaluate from low (left) to high (right) the level of control you exert on your emotional state.

##### 2.5.1.2 Categorical scale

For the categorical scales, we asked participants to evaluate from 0 (not at all) to 100 (totally) the level of anger, frustration, joy, pleasure, sadness, disappointment, relief, and serenity felt. The words were chosen to provide a broad spectrum of different emotions to define their state.

#### 2.5.2 Physiological and ocular measures

ECG, RSP and eye tracking data were collected from participants all along the experiment. For the ECG and RSP signals, the heart/breath rate (HR, BR) and heart/breath rate variabilities (HRV/BRV) measures were used. The HRV and BRV measures included the root mean square of successive difference (RMSSD). HRV measures also included the standard deviation of normal to normal interval (SDNN), the low and high frequencies (LF and HF) and the ratio LF/HF. Eye metrics were relative to the number of fixations on the peripheral (interior and side mirrors) driving environment and the horizontal/vertical gaze variance (HGV, VGV).

### 2.6 Protocol

**Figure 2:**
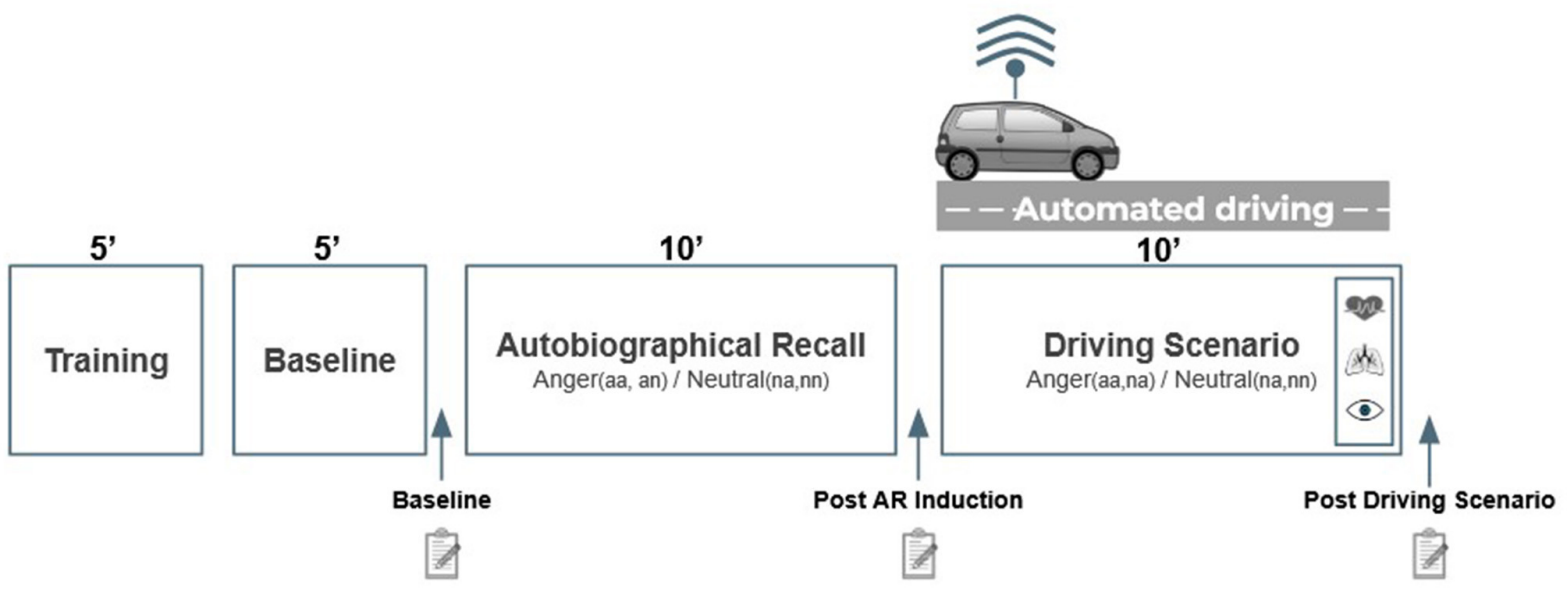
Time course of the experiment. Subjective evaluations are assessed after the baseline, post AR and post DS. Anger effects in physiological and ocular data are only measured from the last minute of the driving scenario.

After participants fulfilled the consent form, we equipped them with all sensors. We then installed them comfortably in the driving simulator cabin and we proceeded to calibrate the eye tracker. The protocol is summarized in Figure 2 and the four steps of training, baseline, AR and DS are documented below.

#### 2.6.1 Training

Participants were trained for ~5 min to drive manually and to switch on/off the autonomous driving mode while respecting system alerts. They were also instructed on how to complete dimensional and categorical emotion questionnaires on the tablet.

#### 2.6.2 Baseline

We first conducted a 5-min rest baseline in which participants were seated in the cabin and instructed to do nothing but keep their eyes open. The baseline is further used for data normalization (see Section 2.7.1).

#### 2.6.3 Autobiographical recall (AR)

The anger/neutral emotion induction by the autobiographical procedure was performed for 10 min (see Section 2.4).

#### 2.6.4 Driving scenario (DS)

Likewise, during the training phase, they were instructed to drive in manual mode while following traffic laws and to switch the autonomous driving mode on and off in response to system alerts. They started in manual mode and quickly the system asked them to give control. During the autonomous drive, four events were manipulated in order to induce anger (see Section 2.4). Moreover, in order to prolong the effect of the emotional induction, they had to constantly think about their emotional experience as long as the vehicle was in autonomous driving mode. The scenario lasted 10 min.

After baseline, AR and DS phases, participants had to report their emotional state by completing the dimensional and categorical emotion assessments.

### 2.7 Data analysis

#### 2.7.1 Data preprocessing

We applied a band-pass filter between 2 and 40 Hz. The Python toolbox Neurokit2 (version 0.2.0; Makowski et al., 2021) and hrv-analysis (version 1.0.4; Champseix et al., 2021) was used in order to find peaks in ECG and RSP signals then calculated corresponding features (see Section 2.5). Because some peaks in ECG may be misplaced or absent (e.g., in case of artifacts caused by movements), all ECG signals were manually checked and peaks were replaced if the algorithm failed to do it. A time window of 60 s without overlapping was used in features calculation.

Delta scores were calculated from subjective (emotion questionnaires) and physiological (ECG, RSP) raw data.

The following transformation was employed for the subjective data:


$$\begin{array}{c} Delta\left(Post\;AR\right)\;=\;Raw\left(Post\;AR\right)\;-\;Raw\left(Baseline\right)\\ Delta\left(Post\;DS\right)\;=\;Raw\left(Post\;DS\right)\;-\;Raw\left(Baseline\right) \end{array}$$

The following transformation was employed for the physiological data (cardiac, respiratory, and electrodermal activities):


$$\begin{array}{c} Delta\left(Last\;minute\;of\;DS\right)\;=\\ \frac{Raw\left(Last\;minute\;of\;DS\right)\;-\;Mean\left(Baseline\right)}{Mean\left(Baseline\right)} \end{array}$$

Concerning eye tracking, raw data were kept for analysis because we did not instruct participants to watch the driving environment during the baseline phase.

### 2.8 Statistical analysis

To determine whether anger induced from different sources (unrelated vs. driving-related) leads to different subjective and physiological responses, we analyzed the subjective and physiological data feature by feature. The subjective data analyzed in this section correspond to the categorical and dimensional emotion assessments completed by participants. Delta scores (difference from baseline) are compared between groups for each moment (Post AR and Post DS) of assessment. For physiological data, delta scores (ratio from baseline) are compared between groups during the last minute of the automated driving scenario. For ocular data, because of the difference between anger and neutral DS, raw scores were compared between the groups *aa*-*na* (anger DS) and *an*-*nn* (neutral DS) during the last minute of the automated driving scenario.

The normality of residuals was not assumed for a large majority of the features explored (checked visually from Q-Q plots and calculated with Shapiro-Wilk statistical tests). Therefore, we used the Kruskal-Wallis test, a non-parametric method enabling us to assess differences between the scores of more than two independent groups. R studio (version 2022.12.0) was used for data analysis. *Post-hoc* comparisons were conducted using Dunn's test with Bonferroni corrections (with *p* < 0.05).

To further explore the relationship between subjective feelings and physiological manifestations, we analyzed correlations by calculating Spearman correlation coefficients. Bonferroni corrections were applied with p < 0.05.

## 3 Results

### 3.1 Subjective evidence

#### 3.1.1 Dimensional scales assessments

**Figure 3:**
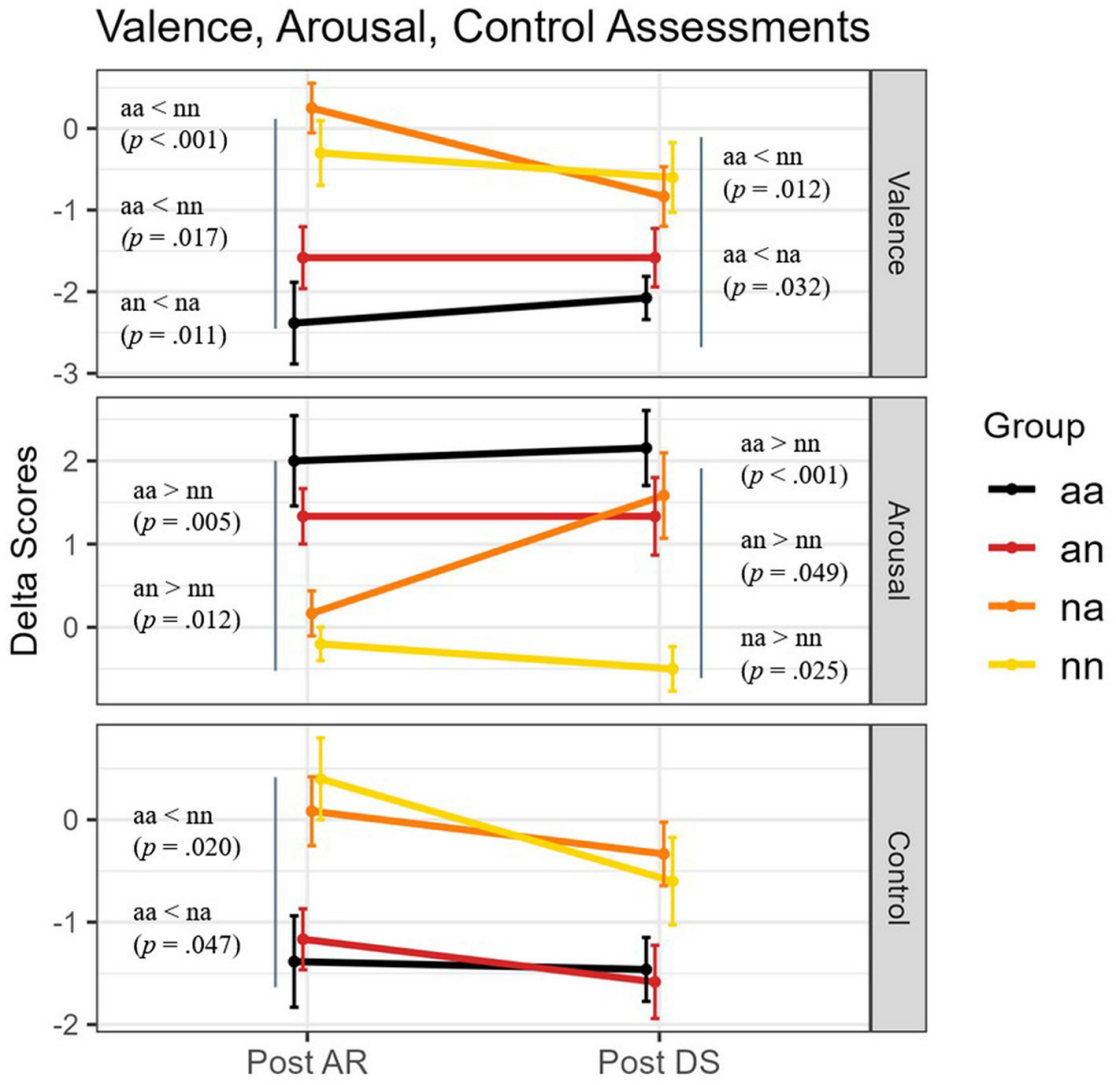
Mean delta scores (difference from baseline) of Valence, Arousal and Control dimensional scale at the different moments (Post AR, Post DS) of the experiment and across the four groups. Error bars represent standard errors. Significant pairwise group comparisons (*p* < 0.05) are reported.

The results are described below and illustrated in Figure 3.

##### Post AR

Kruskal-Wallis test analysis revealed differences among groups for the valence [$\chi_{\left(3\right)}^{2}$ = 21.127, *p* < 0.001, η^2^ = 0.422], arousal [$\chi_{\left(3\right)}^{2}$ = 16.044, *p* < 0.01, η^2^ = 0.303], and control [$\chi_{\left(3\right)}^{2}$ = 13.643, *p* < 0.01, η^2^ = 0.248] dimensions. *Post hoc* test revealed that the score of valence is lower in the *aa* and *an* groups than both *na* and *nn* (*aa-na, Z* = −4.11, *p* < 0.001; *aa-nn, Z* = −2.98, *p* = 0.017; *an-na, Z* = −3.12, *p* = 0.011). The score of arousal is higher for *aa* and *an* groups only in comparison to the *nn* group (*aa-nn, Z* = 3.32, *p* = 0.005; *an-nn, Z* = 3.10, *p* = 0.012). The score of control is lower in *aa* than *nn* and *na* groups (*aa-nn, Z* = −2.94, *p* = 0.020; *aa-na, Z* = −2.66, *p* = 0.047).

##### Post DS

Kruskal-Wallis test analysis revealed differences among groups for the valence [$\chi_{\left(3\right)}^{2}$ = 12.827, *p* < 0.01, η^2^ = 0.229], arousal [$\chi_{\left(3\right)}^{2}$ = 15.292, *p* < 0.01, η^2^ = 0.286], and control [$\chi_{\left(3\right)}^{2}$ = 7.943, *p* < 0.05, η^2^ = 0.115]. *Post hoc* test revealed that the score of valence is lower in the *aa* group compared to the *na* and *nn* groups (*aa-na, Z* = −2.78, *p* = 0.032*; aa-nn, Z* = −3.10, *p* = 0.012). The score of arousal remained significantly higher for *aa, na*, and *an* compared to *nn* (*aa-nn, Z* = 3.78, *p* < 0.001; *na-nn, Z* = 2.87, *p* = 0.025; *an-nn, Z* = 2.65, *p* = 0.049). *Post hoc* tests did not reveal significant differences between groups for the control dimension.

**Figure 4:**
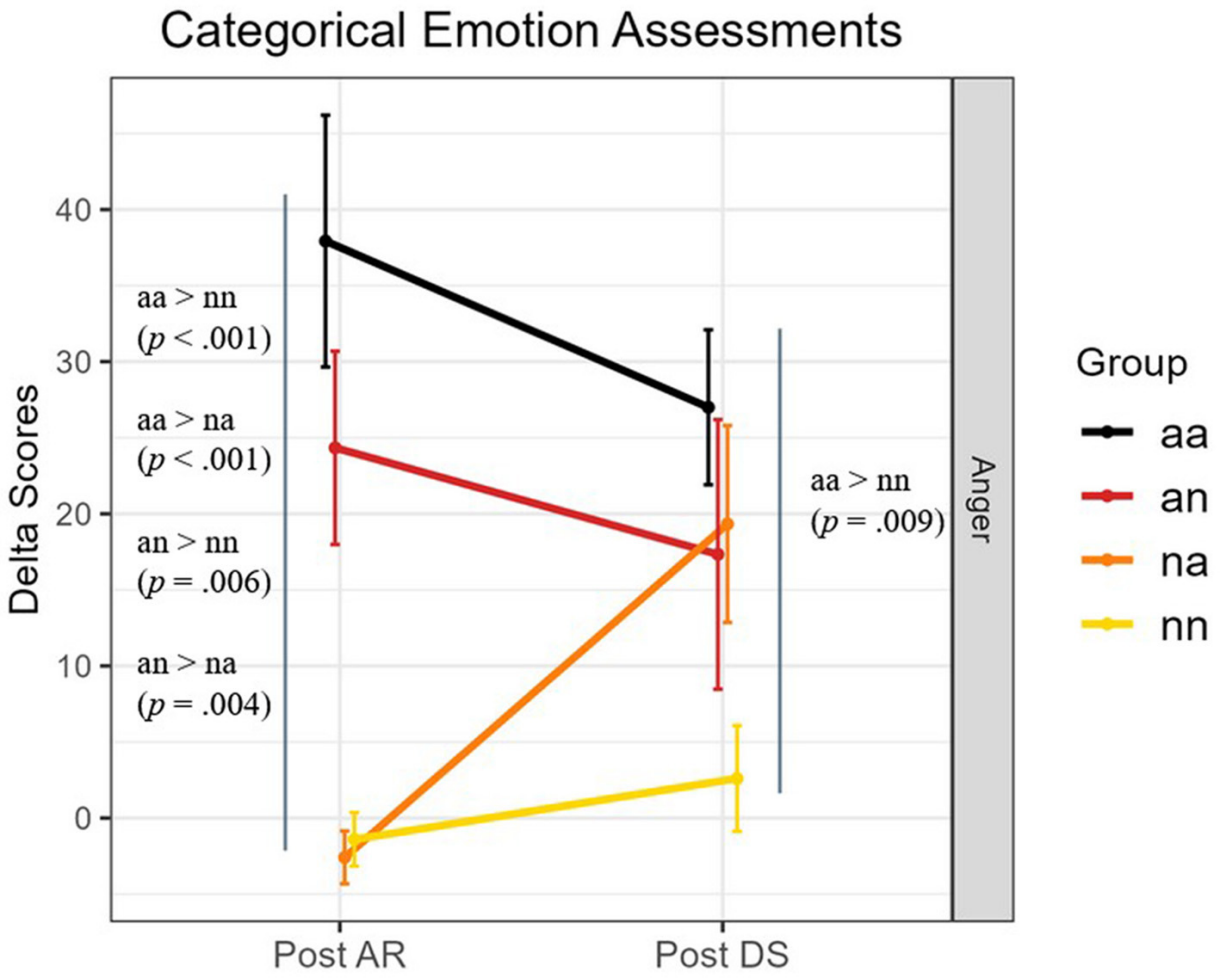
Mean delta scores (difference from baseline) of the Anger emotion scale at the different moments (Post AR, Post DS) of the experiment and across the four groups. Error bars represent standard errors. Significant pairwise group comparisons (*p* < 0.05) are reported.

Because our hypotheses focus solely on the anger score, and in order to facilitate reading, only significant pairwise comparisons about anger evaluation are fully described below and illustrated in Figure 4. Results of Kruskal-Wallis tests for each categorical emotion are provided in the Supplementary material.

#### 3.1.2 Categorical emotion assessments

##### Post AR

Kruskal-Wallis test analysis revealed differences among groups for anger [$\chi_{\left(3\right)}^{2}$ = 32.873, *p* < 0.001, η^2^ = 0.695], frustration [$\chi_{\left(3\right)}^{2}$ =12.467, *p* < 0.01, η^2^ = 0.220], joy [$\chi_{\left(3\right)}^{2}$ =17.202, *p* < 0.001, η^2^ = 0.330], pleasure [$\chi_{\left(3\right)}^{2}$ = 20.388, *p* < 0.001, η^2^ = 0.404], sadness [$\chi_{\left(3\right)}^{2}$ =10.712, *p* < 0.05, η^2^ = 0.179], disappointment [$\chi_{\left(3\right)}^{2}$ =13.488, *p* < 0.01, η^2^ = 0.244], and serenity [$\chi_{\left(3\right)}^{2}$ =9.696, *p* < 0.05, η^2^ = 0.156]. *Post hoc* test revealed that the score of anger is higher in the *aa* and *an* groups than both *na* and *nn* groups (*aa-na, Z* = 4.70, *p* < 0.001; *aa-nn, Z* = 4.16, *p* < 0.001; *an-na, Z* = 3.76, *p* = 0.004; *an-nn, Z* = 3.27, *p* = 0.006).

##### Post DS

Kruskal-Wallis test analysis revealed differences among groups for anger [$\chi_{\left(3\right)}^{2}$ = 10.441, *p* < 0.05, η^2^ = 0.173] and disappointment [$\chi_{\left(3\right)}^{2}$ = 8.652, *p* < 0.05, η^2^ = 0.131]. *Post hoc* test revealed for anger that the difference *aa*-*nn* remained significant (*Z* = 3.17, *p* = 0.009). Nevertheless, the *na* group did not differ significantly (*Z* = 2.27, *p* = 0.140) from the control group.

### 3.2 Physiological and ocular evidence

#### 3.2.1 ECG

Kruskal-Wallis test analysis revealed only differences between groups in HRV_SDNN [$\chi_{\left(3\right)}^{2}$ = 10.357, *p* < 0.05, η^2^ = 0.171]. Nearly significant differences are also observed for HRV_LF [$\chi_{\left(3\right)}^{2}$ = 7.477, *p* = 0.058, η^2^ = 0.104]. *Post hoc* tests revealed that the *aa* group showed an increase in SDNN compared to *an* (*Z* = 2.89, *p* = 0.023).

No significant results were obtained for HR, BR, LF/HF, HF, HRV_RMSSD (*ps* > 0.150, see Supplementary material).

#### 3.2.2 RSP

Kruskal-Wallis test analysis revealed nearly significant differences between groups in BRV_RMSSD [$\chi_{\left(3\right)}^{2}$ = 7.749, *p* = 0.052, η^2^ = 0.110]. The score of RMSSD tended (*Z* = −2.56, *p* = 0.063) to be higher for the *nn* group than the *na* group.

#### 3.2.3 Eye tracking

Neither Kruskal-Wallis tests of VGV, HGV and the number of fixations on mirrors reached significance for the *aa*-*na* and *an*-*nn* comparisons (*ps* > 0.217, see Supplementary material).

**Figure 5:**
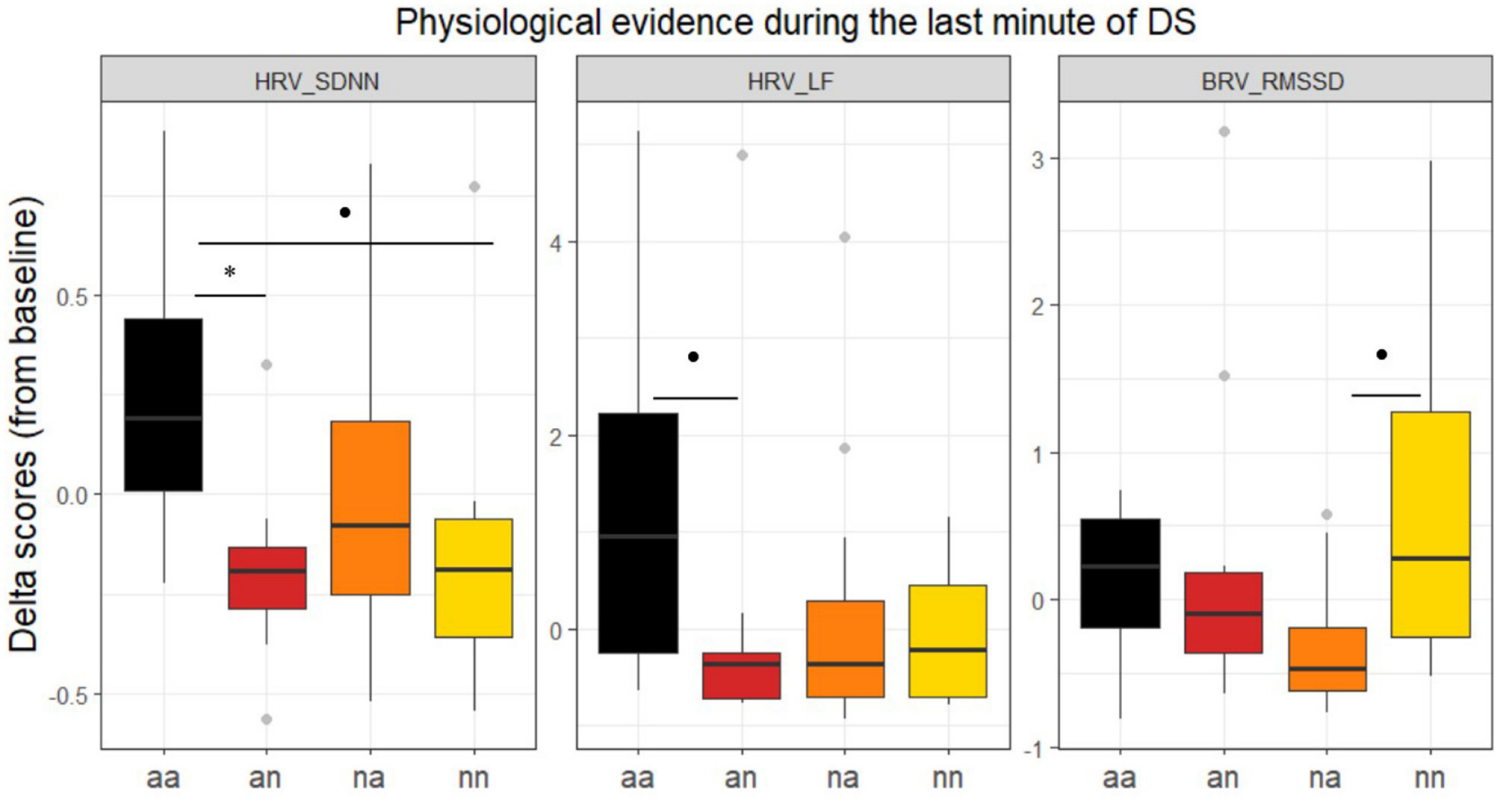
Delta scores (rapport from baseline) across groups for HRV_SDNN, HRV_LF and BRV_RMSSD during the last minute of the driving scenarios. Error bars represent standard errors. *p < 0.05; . < 0.10.

Significant results are illustrated in Figure 5 and summarized in Table 3.

**Table 3:** Summary of significant results of Kruskal-Wallis tests regarding subjective (valence, arousal, control, anger), cardiac, respiratory, and eye tracking data.

**Indicators**	**Moment**	**χ^2^**	***p***	**η^2^**	**Group differences**
**Subjective**					
Anger	Post AR	32.873	<0.001	0.695	aa > nn (*Z* = 4.16, *p* < 0.001) aa > na (*Z* = 4.70, *p* < 0.001) an > nn (*Z* = 3.27, *p* = 0.006) an > na (*Z* = 3.76, *p* = 0.004)
	Post DS	10.441	<0.05	0.173	aa > nn (*Z* = 3.17, *p* = 0.009)
Arousal	Post AR	16.044	<0.01	0.303	aa > nn (*Z* = 3.32, *p* = 0.005) an > nn (*Z* = 3.10, *p* = 0.012)
	Post DS	15.292	<0.01	0.286	aa > nn (*Z* = 3.78, *p* < 0.001) an > nn (*Z* = 2.65, *p* = 0.049) na > nn (*Z* = 2.87, *p* = 0.025)
Valence	Post AR	21.127	<0.001	0.422	aa < na (*Z* = −4.11, *p* < 0.001) an < na (*Z* = −3.12, *p* = 0.011) aa < nn (*Z* = −2.98, *p* = 0.017)
	Post DS	12.827	<0.01	0.229	aa < nn (*Z* = −3.10, *p* = 0.012) aa < na (*Z* = −2.78, *p* = 0.032)
Control	Post AR	13.643	<0.01	0.248	aa < nn (*Z* = −2.94, *p* = 0.020) aa < na (*Z* = −2.66, *p* = 0.047)
	Post DS	7.944	<0.05	0.115	
**Cardiac**					
HRV_SDNN	Last minute of DS	10.357	<0.05	0.171	aa > an (*Z* = 2.89, *p* = 0.023)
HRV_LF	Last minute of DS	7.477	0.058	0.104	
**Respiration**					
BRV_RMSSD	Last minute of DS	7.749	0.052	0.110	

### 3.3 Correlations analysis between subjective, physiological, and ocular data

Spearman correlations analysis were performed to examine the relationships between subjective reports and physiological variables (Table 4). Only strong correlations are observed within subjective and cardiac data. Notably, the arousal dimension was positively correlated with anger (rho = 0.55; *p* < 0.01) and negatively correlated with valence (rho = −0.49; *p* < 0.05). Valence is positively correlated with control (rho = 0.61; *p* < 0.001). HRV_SDNN was positively correlated with HRV_LF (rho = 0.64; *p* < 0.001) and HRV_RMSSD (rho = 0.60; *p* < 0.001). HRV_LF was also positively correlated with HRV_LF/HF (rho = 0.79; *p* < 0.001) and HRV_RMSSD with HRV_HF (rho = 0.57; *p* < 0.01). However, no significant correlations were observed between subjective and physiological measures.

**Table 4:** Spearman correlations between subjective and physiological variables.

	**Variables**									
**Variables**	**1**	**2**	**3**	**4**	**5**	**6**	**7**	**8**	**9**	**10**
1. Anger										
2. Valence										
3. Arousal	0.55	−0.49								
4. Control		0.61								
5. HRV_SDNN										
6. HRV_LF					0.64					
7. HRV_LF/HF						0.79				
8. HRV_RMSSD					0.60					
9. HRV_HF								0.57		
10. BRV_RMSSD										

## 4 Discussion

Anger is prevalent in driving contexts (Underwood et al., 1999) and can arise from both on-road incidents and external influences, such as sudden cut-offs (Wickens et al., 2013) and negative thought rumination (Suhr, 2016). Given the well-established positive correlation between anger and aggressive driving behaviors (Bogdan et al., 2016), monitoring the driver's emotional state is crucial to enhance road safety. To effectively detect and mitigate anger during driving, it is imperative to understand how anger, originating from both driving environments and external factors, affects physiological responses and visual strategies. This study aimed to explore these dynamics, focusing on how the individual and combined effects of different anger sources impact subjective, physiological responses and visual behavior in driving context. Such understanding is essential to guide the development of adaptive in-car systems and improve driver safety.

We investigated two distinct sources of anger: non-driving-related anger induced via Autobiographical Recall (AR) and anger triggered by driving-related events during automated driving (Driving Scenario, DS). By employing automated driving, motor activity's influence on physiological signals was minimized, ensuring a clearer assessment of emotional responses. AR simulated rumination of negative thoughts while DS involved realistic scenarios that could provoke anger during driving. Each induction was followed by measurements using subjective reports (dimensional and categorical scales). The physiological data (ECG, RSP), and ocular behavior from the last minute of the driving scenario were compared between groups to assess anger's impact comprehensively.

The results obtained here demonstrated that combined anger sources elicited more pronounced emotional and physiological responses than either source individually. Specifically, no significant physiological and ocular differences were observed between the two sources when tested separately; however, their subjective emotional impacts varied. AR induced a wider range of negative emotions, including frustration and sadness, while DS revealed large individual variability in anger intensity.

After discussing the effectiveness of the anger induction techniques used in this study, the subjective, physiological, and ocular evidence highlighted in the results is discussed below and some implications are given for the development of anger control and regulation systems.

### 4.1 Effectiveness of AR and DS induction

The effectiveness of AR in inducing anger was consistent with literature findings (e.g., Jallais and Gilet, 2010). Participants reported high arousal, alongside reduced valence and control immediately after AR induction. Interestingly, while anger was the dominant emotion, other negative feelings such as sadness and frustration also emerged, consistent with (Mills and D'Mello, 2014). One interesting finding concerns the differences between groups *an*-*aa* and *an*-*nn*. In *an* and *aa*, participants were first induced in anger by AR and reported (in post AR measurement) an increase in anger and arousal alongside a reduced valence. In the *an* group (confronted to neutral DS), the anger and valence modulations faded over time (in the post DS measurement) while the increase in arousal remained. In the control group (*nn*), no emotional modulation is observed. This suggests that AR differently modulates anger, valence, and arousal over time. Valence and anger are modulated over a short period, while arousal is modulated over a longer period. Therefore, studies that induce anger using AR and assess its effects solely through anger levels (e.g., FakhrHosseini and Jeon, 2019) should also consider arousal and valence levels to provide a more comprehensive understanding. Additionally, future research should investigate the duration of anger and valence post-induction to better understand their temporal dynamics. To prolong the emotional impact of AR-induced anger, future studies might benefit from integrating emotionally charged music alongside AR (Braun et al., 2018; Steinhauser et al., 2018) to sustain the induced emotional state for longer periods.

For the DS, incorporating specific impeding events caused minor increases in anger, arousal, and disappointment. Notably, no modulation of valence was observed. While AR effectively reduced valence levels at short-term, DS alone (in the *na* group) did not produce the same effect. This finding should be considered alongside the fact that AR induced sadness, an emotion with negative valence, that DS did not trigger. Thus, although both techniques influenced anger and arousal levels, only AR affected the valence dimensions. Moreover, DS introduced more variability in anger levels than AR did. This variability echoes the findings of Cazes et al. (2024) on the different profiles of drivers in autonomous driving. Faced with the same situations on the road, participants' reactions differ, ranging from those wishing to take control at the slightest complication to those letting the system handle everything as long as there are no alerts. We therefore believe that the different anger reactions of our participants are linked to these differences in profiles. Those who felt little or no anger in the angry driving scenario may be those who don't want to be in control of the vehicle, letting the system handle any situation. Conversely, those with high anger scores may present a profile of drivers wanting to regain control at all costs in any situation. We recommend that future studies on the autonomous driving paradigm integrate the dimension of situational control, by assessing, for example, the level of trust in automation (Körber et al., 2018) and the locus of control (Özkan and Lajunen, 2005). Drivers with high takeover willingness should be a focus of further study, as they are prone to more aggressive behaviors during takeovers (Pan et al., 2024).

The differences observed in the emotions felt after AR and DS inductions resonate with the study of Parkinson's (2001). Responses from questionnaires highlighted that anger experienced while driving tends to be less intense but more distinct, with fewer emotional blends, compared to anger unrelated to the driving activity. The dimension of valence thus seems to be decisive in understanding the source of anger. Research by Du et al. (2020) suggests that emotional valence, regardless of arousal level, significantly affects takeover performance in automated driving. In our correlation analysis, the level of emotional valence was not correlated with any of the physiological indicators investigated in this study. This analysis was made between subjective measures taken at post DS and physiological measures taken from the last minute of DS. However, we have previously discussed that valence modulation seems to occur in the short term after AR induction. Thus, while changes in emotional valence influence driving performance, they may not be easily captured by heart/breath physiological measures. To enhance the sensitivity and accuracy of in-vehicle emotional monitoring systems, it is crucial to incorporate additional indicators that can capture emotional valence. We suggest that future studies explore the use of facial expression analysis as a promising approach. Recent advancements in deep learning, such as those reported by Toisoul et al. (2021), have shown encouraging results in detecting subtle changes in facial expressions.

### 4.2 Evidence from physiological data

Our findings do not support the commonly reported elevation in HR found in previous literature (e.g., FakhrHosseini and Jeon, 2019). Instead, they align with studies that report no significant effects (e.g., Wang et al., 2024). However, HRV data underscored autonomic nervous system activation. The double-induced group presented increased heart rate variability (SDNN, LF (nearly-significant) values) without any change in RMSSD and HF values. These differences were particularly significant for SDNN when comparing the *aa* group with the *an* group.

SDNN serves as a global measure of long-term sympathetic and parasympathetic activity, while RMSSD and HF primarily reflect parasympathetic modulation, and LF is more indicative of sympathetic activation (Li and Zheng, 2022). Correlation analyses in our study revealed strong associations between SDNN and LF, SDNN and RMSSD, and SDNN and LF/HF. However, no significant correlations were found between subjective emotional responses and physiological measures. The relationship between anger, and SDNN remains debated in the literature. Some studies have reported an increase in SDNN following DS induction (Wang et al., 2024), while others have found no effect of AR induction on SDNN (FakhrHosseini and Jeon, 2019). In our study, neither AR nor DS alone significantly modulated SDNN, but their combination did. On average, anger scores increased by almost 20 points (out of 100) for the *an* and *na* groups, and by almost 30 for the *aa* group. In Wang et al. (2024) participants reported raw anger scores between 49 and 76 in relation to the events in the driving scenario. This suggests that anger may require a certain intensity threshold before SDNN changes become evident. The observed change may also be linked to regulatory strategies implemented by the participants. Indeed, emotional regulation strategies could influence SDNN outcomes. For instance, Francis et al. (2015) found that SDNN increased following anger induction via arithmetic tasks and video clips, but rather for participants who had previously taken part in a biofeedback regulation exercise. Further research is needed to clarify the relationship between anger and HRV. To do this, it would be interesting to propose a regulation exercise to the *aa* and *nn* groups and compare their SDNN values with the initial groups.

Respiratory data revealed that the *na* group exhibited near-significant lower RMSSD value compared to the *nn* group. This supports findings from Ritsert et al. (2022) and Soni and Muniyandi (2019), which noted higher RMSSD levels in relaxed individuals or meditators. This marker could be worth exploring further to dynamically measure the effectiveness of regulation aimed at calming an angry state.

Taken together, cardiac and respiratory results suggest that anger predominantly activates the sympathetic system. However, because physiological indicators of anger are only visible in the double anger induced group, this also suggests that a feeling of anger is not always associated with detectable physiological manifestations.

According to Scherer's Component Process Model (Scherer, 2009), emotions are dynamic states. They result from continuous, multi-level evaluations of a situation. During the autobiographical recall exercise, individuals might recount events where they felt intense anger in the past but now have come to terms with those experiences. As a result, the initial anger may be less aroused and nuanced with less intense negative emotions, such as sadness or disappointment. This could explain why lower levels of felt anger do not always correspond with clear physiological manifestations. For the DS induction, instead of looking at the difference before/after, continuous monitoring of physiological signals can provide a more nuanced understanding of emotional changes in an angry detection model (Yan et al., 2018).

### 4.3 Evidence from ocular data

Contrary to initial expectations and the literature, angry participants did not display a narrowing of visual attention. This lack of effect may be attributed to autonomous driving when environment supervision is required. However, in the study of Pan et al. (2024) this narrowing of the visual field was also measured in autonomous driving requiring supervision. The most likely interpretation is related to trust in automation. Previous studies (Hergeth et al., 2016; Körber et al., 2018) suggested that higher trust correlates with lower road monitoring. Participants in Pan et al. (2024) were taxi drivers and perhaps they present higher trust toward automation than our participants. This highlights the need for further exploration of the relationship between anger expression and trust in automation.

### 4.4 Implications for anger detection and regulation systems

Our findings suggest that the persistence of anger in the *aa* group may reflect a threshold effect where, once a certain level of anger is reached, anger is sustained with subsequent irritating events. The emergence of anger as an emotion reflects a dynamic process (Scherer, 2009), often preceded by related negative states such as frustration (Bosch et al., 2020).

Our results have important implications for managing anger in driving contexts. Preemptive strategies, such as mindfulness exercises or creating a calming vehicle environment, should be used to help drivers avoid reaching this threshold. However, when anger crosses this threshold and becomes entrenched, longer-term interventions, such as cognitive approaches (e.g., reappraisal, Harris and Nass, 2011), or strong behavioral strategies that switch attention away (in autonomous driving) may be necessary.

### 4.5 Limitations and future directions

Our study faced limitations, notably a small sample size that restricted analysis of individual differences. Additionally, conducting the study in a driving simulator may not fully replicate real-world driving's complexity and stressors. Future research should aim for larger samples and incorporate real-world driving tests to validate these findings. Anger traits were not assessed in this study but could influence individual variability. The inclusion of the Anger Rumination Scale (ARS; Sukhodolsky et al., 2001) and the Driving Anger Scale (DAS; Deffenbacher et al., 1994) in future research could help clarify these individual differences. Moreover, assessing driver profiles based on locus of control (e.g., multidimensional traffic locus of control; Özkan and Lajunen, 2005) and trust in automation (e.g., Körber, 2019) could further refine our understanding of anger's impact on different driver types.

For the development of driver monitoring systems, the user experience factors must be considered. Failures in automated driving systems have been shown to reduce trust and positive experiences, ultimately influencing willingness to use automated vehicles (Liu et al., 2021). People generally agree that their mental state should be monitored in the vehicle (Smyth et al., 2021), but a main concern is that the system is too inaccurate to detect anger (Li S. et al., 2020). To optimize in-vehicle anger regulation systems, we recommend that future in-car systems assess drivers' emotional states upon entering the vehicle rather than solely in response to road events.

For researchers, we encourage to employ a mix of methods to most effectively induce an angry state. To further understand the evolution of anger in this interplay, we recommend additionally assessing the dimensions of valence and arousal, and monitoring the emotional state after each annoying driving event as proposed in Wang et al. (2024).

### 4.6 Conclusion

To conclude, while anger induced by driving-related events and autobiographical recall yielded distinct subjective emotional responses, physiological, and ocular responses were similar when analyzed separately. The combination of both sources proved more effective at eliciting and sustaining anger, as evidenced by heightened subjective and physiological changes. These findings emphasize that anger in driving contexts is often the result of cumulative effects, where pre-existing anger can amplify responses to frustrating road events. Rather than focusing on differentiating between sources of anger, systems designed for detecting and managing anger should consider the overall dynamics of contributing factors. Future research should prioritize examining these interactions in real-world settings to validate these findings and optimize in-car systems for better emotional monitoring and regulation support.

## Data Availability

The datasets presented in this article are not readily available because the participants of this study did not give written consent for their data to be shared publicly, so due to the sensitive nature of the research supporting data is not available. Requests to access the datasets should be directed to jordan.maillant@valeo.com.
