# Supplementary material for: Detecting sources of anger in automated driving: driving-related and external factor
Source: Front Neuroergon. 2025 May 9;6:1548861. doi: 10.3389/fnrgo.2025.1548861 (PMC12098279; doi:10.3389/fnrgo.2025.1548861)
Supplement: Supplementary file 1 [file Table_1.docx]

***Supplementary Material***

# Supplementary Tables

Table 1. A detailed description of the four driving events depicted in both the anger and neutral driving scenarios. Pictures illustrate the behavior of vehicles in the anger scenario. The participants' vehicle (referred to as the "ego vehicle") is yellow, while obstructing vehicles are yellow, and non-obstructing vehicles are black.

| Event ID | Duration (s) | Descriptions: anger and neutral scenarios |
| --- | --- | --- |
| **1.**  **Tailgating**  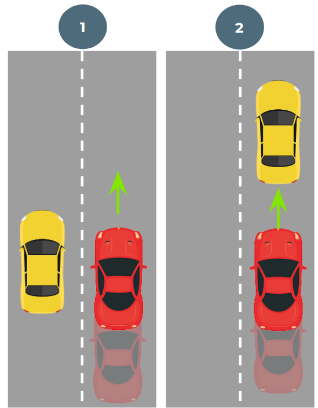 | 60 | *Anger scenario:*  The ego vehicle attempts to overtake another car, but the latter accelerates.  Despite this, the ego vehicle successfully overtakes. However, after being passed, the other vehicle quickly closes the gap, tailgates the ego vehicle, overtakes it again, and speeds away at high velocity. |
|  |  | *Neutral scenario:*  The ego vehicle overtakes another car. |
| **2.**  **Slow vehicle**  **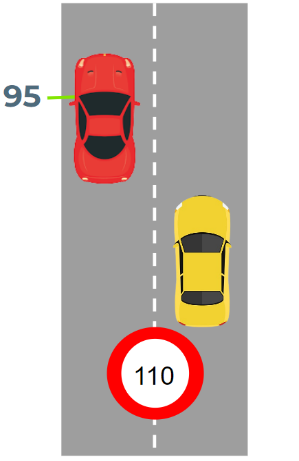** | 60 | *Anger scenario:*  The ego vehicle is driving in the right lane. It slows down as another vehicle moves slowly into the left lane. Overtaking from the right is not allowed under the highway code. The situation continues until the ego vehicle moves to a freeway fork. |
|  |  | *Neutral scenario:*  The ego vehicle is driving in the right lane. It slows down as the speed limit decreases. There is another vehicle ahead in the right lane. |
| **3.**  **Emergency lane**  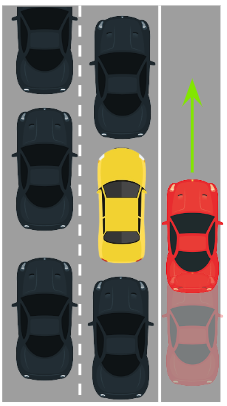 | 30 | *Anger scenario:*  The ego vehicle is driving slowly because it's stuck in traffic. Another vehicle overtakes everyone in the emergency lane. |
|  |  | *Neutral scenario:*  The ego vehicle is driving slowly because it's stuck in traffic. An ambulance overtakes everyone in the emergency lane. |
| **4.**  **Staying right**  **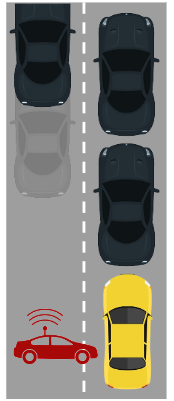** | 30 | *Anger scenario:* The traffic jam clears, but first only in the left-hand lane. The ego vehicle does not decide to overtake and it remains on the right until the traffic clears. |
|  |  | *Neutral scenario:* The traffic jam clears on both lanes. |

Table 2. Results of Kruskal-Wallis tests for each delta score of emotion assessed at post Autobiographical Recall (AR) and Post Driving Scenario (DS).

| **Indicators** | **Moment** | **Kruskal-Wallis test** | | | **Median**  **(IQR)** | | | | **Group differences** |
| --- | --- | --- | --- | --- | --- | --- | --- | --- | --- |
|  |  | *χ2* | *p* | *η2* | *aa* | *an* | *na* | *nn* | *Bonferroni (alpha ≤ .05)* |
| **Arousal** | Post AR | 16.044 | <.01 | .303 | 2.00  (4.00) | 1.00  (1.25) | 0.00  (0.25) | 0.00  (0.75) | aa > nn (Z = 3.32, *p* = .005)  an > nn (Z = 3.10, *p* = .012) |
|  | Post DS | 15.292 | <.01 | .286 | 2.00  (3.00) | 1.00  (2.00) | 1.00  (3.00) | -0.50  (1.00) | aa > nn (Z = 3.78, *p* < .001)  an > nn (Z = 2.65, *p* = .049)  na > nn (Z = 2.87, *p* = .025) |
| **Valence** | Post AR | 21.127 | <.001 | .422 | -2.00  (2.00) | -1.50  (1.50) | 0.00  (0.25) | 0.00  (0.75) | aa < na (Z = -4.11, *p* < .001)  an < na (Z = -3.12, *p* = .011)  aa < nn (Z = -2.98, *p* = .017) |
|  | Post DS | 10.441 | <.0.5 | .229 | -2.00  (2.00) | -1.00  (1.25) | 0.00  (1.25) | 0.00  (1.00) | aa < nn (Z = -3.10, *p* = .012)  aa < na (Z = -2.78, *p* = .032) |
| **Control** | Post AR | 13.643 | <.01 | .248 | -2.00  (1.00) | -1.00  (2.00) | 0.00  (0.50) | 0.00  (0.75) | aa < nn (Z = -2.94, *p* = .020)  aa < na (Z = -2.66, *p* = .047) |
|  | Post DS | 7.944 | <.05 | .115 | -2.00  (2.00) | -1.50  (2.25) | 0.00  (1.00) | -1.00  (1.75) |  |
| **Anger** | Post AR | 32.873 | <.001 | .695 | 30.00  (44.00) | 17.50  (41.25) | 0.00  (2.75) | 0.00  (0.00) | aa > nn (Z = 4.16, *p* < .001)  aa > na (Z = 4.70, *p* < .001)  an > nn (Z = 3.27, *p* = .006)  an > na (Z = 3.76, *p* = .004) |
|  | Post DS | 10.441 | <.05 | .173 | 27.00  (18.00) | 3.50  (36.00) | 12.50  (32.00) | 0.00  (0.00) | aa > nn (Z = 3.17, *p* = .009) |
| **Frustration** | Post AR | 12.467 | <.01 | .220 | 25.00  (24.00) | 5.50  (26.25) | 0.00  (1.50) | 0.00  (9.25) | aa > na (Z = 3.24, *p* < .01) |
|  | Post DS | 1.962 | .580 |  | 14.00  (31.00) | 19.50  (36.25) | 0.00  (44.00) | 3.00  (12.50) |  |
| **Joy** | Post AR | 17.202 | <.001 | .330 | -27.00  (36.00) | -9.50  (26.00) | -0.50  (13.25) | 4.50  (15.75) | aa < nn (Z = -3.73, *p* < .01)  an < nn (Z = -2.79, *p* < .05)  aa < na (Z = -2.78, *p* < .05) |
|  | Post DS | 7.722 | .052 |  | -16.00  (20.00) | -18.00  (22.25) | 0.00  (8.25) | -4.50  (21.25) |  |
| **Pleasure** | Post AR | 20.388 | <.001 | .404 | -33.00  (11.00) | -13.50  (17.75) | 0.00  (12.00) | 0.00  (5.50) | aa < nn (Z = -3.30, *p* < .01)  aa < na (Z = -4.07, *p* < .001) |
|  | Post DS | 7.699 | .053 |  | -27.00  (36.00) | -29.50  (28.25) | -3.50  (15.50) | -13.50  (27.75) |  |
| **Sadness** | Post AR | 10.712 | <.05 | .179 | 33.00  (58.00) | 8.50  (39.50) | 1.00  (6.00) | 0.00  (0.00) | aa > nn (Z = 3.05, *p* < .05) |
|  | Post DS | 7.583 | .055 |  | 11.00  (26.00) | 19.50  (60.00) | 0.00  (3.25) | 0.00  (0.00) |  |
| **Disappointment** | Post AR | 13.488 | <.01 | .244 | 14.00  (63.00) | 1.50  (37.25) | 0.00  0.25 | 0.00  (7.25) | aa > nn (Z = 3.34, *p* < .01) |
|  | Post DS | 8.652 | <.05 | .131 | 14.00  (24.00) | 4.00  (26.75) | 0.00  (0.25) | 0.00  (10.00) |  |
| **Relief** | Post AR | 0.916 | 0.82 |  | -15.00  (42.00) | -1.00  (5.75) | -3.00  (20.00) | -10.50  (19.75) |  |
|  | Post DS | 5.230 | .156 |  | -16.00  (49.00) | -10.00  (23.00) | -2.00  (21.25) | 0.50  (11.00) |  |
| **Serenity** | Post AR | 9.696 | <.05 | .156 | -28.00  (34.00) | -13.50  (32.50) | 0.00  (4.25) | 0.00  (11.00) |  |
|  | Post DS | 4.800 | .187 |  | -17.00  (34.00) | -16.00  (33.00) | -10.00  (31.25) | -2.5  (16.00) |  |

Table 3. Results of Kruskal-Wallis tests for physiological measures assessed during the last minute of the Driving Scenario (DS)

| **Indicators** | **Kruskal-Wallis test** | | | **Median**  **(IQR)** | | | | **Group differences** |
| --- | --- | --- | --- | --- | --- | --- | --- | --- |
|  | *χ2* | *p* | *η2* | *aa* | *an* | *na* | *nn* | *Bonferroni (alpha ≤ .05)* |
| **Cardiac** |  |  |  |  |  |  |  |  |
| HR | 1.338 | .720 |  | 0.00  (0.08) | 0.02  (0.07) | 0.00  (0.05) | 0.01  (0.04) |  |
| HRV_SDNN | 10.357 | <.05 | .171 | 0.19  (0.43) | -0.19  (0.15) | -0.08  (0.44) | -0.19  (0.29) | aa > an (Z = 2.89, *p* = .023) |
| HRV_LF | 7.477 | .058 |  | 0.96  (2.47) | -0.37  (0.48) | -0.37  (0.99) | -0.22  (1.16) |  |
| HRV_HF | 4.311 | .023 |  | -0.05  (1.00) | -0.41  (0.28) | 0.05  (0.69) | -0.25  (0.40) |  |
| HRV_LF/HF | 3.089 | .378 |  | 0.21  (4.46) | -0.09  (1.29) | -0.28  (0.88) | 0.41  (1.69) |  |
| HRV_RMSSD | 5.321 | .150 |  | 0.04  (0.19) | -0.30  (0.58) | -.010  (0.27) | -0.04  0.39 |  |
| **Respiration** |  |  |  |  |  |  |  |  |
| BR | 1.314 | .726 |  | 0.07  (0.25) | 0.08  (0.20) | 0.12  (0.17) | 0.06  (0.18) |  |
| BRV_RMSSD | 7.749 | .052 |  | 0.23  (0.74) | -0.10  (0.54) | -0.47  (0.43) | 0.28  (1.54) |  |

Table 4. Results of Kruskal-Wallis tests for ocular measures assessed during the last minute of the Driving Scenario (DS). Comparisons were between *aa-na* and *an-nn* groups.

| **Indicators** | Groups compared | **Kruskal-Wallis test** | | **Median**  **(IQR)** | | | |
| --- | --- | --- | --- | --- | --- | --- | --- |
|  |  | *χ2* | *p* | *aa* | *an* | *na* | *nn* |
| VGV | aa-na | 1.227 | .268 | 212.74  (143.21) | 197.88  (157.08) | 184.85  (61.47) | 191.90  (86.35) |
|  | an-nn | 0.526 | .468 |  |  |  |  |
| HGV | aa-na | .034 | .854 | 565.55  (196.85) | 554.15  (364.83) | 563.76  (267.55) | 521.36  (96.80) |
|  | an-nn | 0.735 | .391 |  |  |  |  |
| Number of fixations on mirrors | aa-na | 1.522 | .217 | 45.00  (5.00) | 35.00  (10.25) | 36.50  (13.25) | 31.50  (14.25) |
|  | an-nn | 0.529 | .467 |  |  |  |  |
